# Determinants of orthopedic physicians’ self-reported compliance with surgical site infection prevention: results of the WACH-trial’s pilot survey on COM-B factors in a German university hospital

**DOI:** 10.1186/s13756-021-00932-9

**Published:** 2021-04-07

**Authors:** Ivonne Tomsic, Ella Ebadi, Frank Gossé, Ina Hartlep, Pamela Schipper, Christian Krauth, Bettina Schock, Iris F. Chaberny, Thomas von Lengerke

**Affiliations:** 1 Centre for Public Health and Healthcare, Department of Medical Psychology (OE 5430), Hannover Medical School, Carl-Neuberg-Str. 1, 30625 Hannover, Germany; 2 Centre for Laboratory Medicine, Institute of Medical Microbiology and Hospital Epidemiology, Hannover Medical School, Hannover, Germany; 3 Spinal Surgery and Conservative Orthopaedics, Clinic of Orthopaedics of Hannover Medical School at DIAKOVERE Annastift, Hannover, Germany; 4 Center for Infection Medicine (ZINF), Institute of Hygiene, Hospital Epidemiology and Environmental Medicine, Leipzig University Hospital, Leipzig, Germany; 5 Centre for Public Health and Healthcare, Institute of Epidemiology, Social Medicine and Health Systems Research, Hannover Medical School, Hannover, Germany

**Keywords:** Surgical site infections, Guideline adherence, Orthopedics, Surgery, Physicians, Behavior change

## Abstract

**Background:**

Prevention of surgical site infections (SSIs), which due to their long-term consequences are especially critical in orthopedic surgery, entails compliance with over 20 individual measures. However, little is known about the psychosocial determinants of such compliance among orthopedic physicians, which impedes efforts to tailor implementation interventions to improve compliance. Thus, for this professional group, this pilot survey examined psychosocial determinants of self-reported compliance, which have been theoretically derived from the COM-B (Capability, Opportunity, Motivation and Behavior) model.

**Methods:**

In 2019, a cross-sectional survey was conducted in a tertiary care university orthopedic clinic in Hannover, Germany, as a pilot for the WACH-trial (“Wundinfektionen und Antibiotikaverbrauch in der Chirurgie” [Wound Infections and Antibiotics Consumption in Surgery]). Fifty-two physicians participated (38 surgeons, 14 anesthesiologists; response rate: 73.2%). The questionnaire assessed self-reported compliance with 26 SSI preventive measures, and its psychosocial determinants (COM-B). Statistical analyses included descriptive, correlational, and linear multiple regression modeling.

**Results:**

Self-reported compliance rates for individual measures varied from 53.8 to 100%, with overall compliance (defined for every participant as the mean of his or her self-reported rates for each individual measure) averaging at 88.9% (surgeons: 90%, anesthesiologists: 85.9%; *p* = 0.097). Of the components identified in factor analyses of the COM-B items, planning, i.e., self-formulated conditional plans to comply, was the least pronounced (mean = 4.3 on the 7-point Likert scale), while motivation was reported to be the strongest (mean = 6.3). Bi-variately, the overall compliance index co-varied with all four COM-B-components, i.e., capabilities (r = 0.512, *p* < 0.001), opportunities (r = 0.421, *p* = 0.002), planning (r = 0.378, *p* = 0.007), and motivation (*r* = 0.272, *p* = 0.051). After mutual adjustment and adjustment for type of physician and the number of measures respondents felt responsible for, the final backward regression model included capabilities (β = 0.35, *p* = 0.015) and planning (β = 0.29, *p* = 0.041) as COM-B-correlates.

**Conclusion:**

Though based on a small sample of orthopedic physicians in a single hospital (albeit in conjunction with a high survey response rate), this study found initial evidence for positive correlations between capabilities and planning skills with self-reported SSI preventive compliance in German orthopedic physicians. Analyses of the WACH-trial will further address the role of these factors in promoting SSI preventive compliance in orthopedic surgery.

*Trial registration*: This survey was conducted as part of the research project WACH ("Wundinfektionen und Antibiotikaverbrauch in der Chirurgie" [Wound Infections and Antibiotic Consumption in Surgery]), which has been registered in the German Clinical Trial Registry (https://www.drks.de/; ID: DRKS00015502).

**Supplementary Information:**

The online version contains supplementary material available at 10.1186/s13756-021-00932-9.

## Introduction

Surgical site infections (SSIs) occur worldwide and represent a common nosocomial infection [1, 2]. For instance, in Germany, 22.4% of all nosocomial infections in 2016 were SSIs [3]. SSIs can lead to increased length of hospital stay, morbidity and mortality rates, and healthcare costs [4–6]. While they occur in all surgery fields, they are especially critical in orthopedic surgery [6]. Several evidence-based measures have been recommended to prevent SSIs [7]. While studies show that a significant number could be avoided by the correct implementation of measures [8, 9] the latter are not always compliantly implemented [10].

Therefore, appropriate behavior changes among health care professionals are still necessary. In this context, different implementation interventions are available to promote compliance [11, 12]. Even though there exist several types of such implementation interventions [12], more often than not strategies with a focus on standard interventions such as education or reminders are used [13, 14]. More critically, implementation interventions are often not chosen on the basis of previous analysis and/or theory but are rather selected because they have been used before or are familiar to the healthcare professional [15]. However, regarding behavior change and compliance promotion, tailored interventions, i.e., “…strategies to improve professional practice…taking into account of prospectively identified determinants of practice…” [16, p. 5] have been shown to be more effective than standard, one-size-fits-all strategies [16–18]. Thus, it is necessary to capture what exactly has to be changed in order to successfully promote compliance and eventually reduce SSI incidence [19].

However, little is known about the determinants of compliance with SSI preventive measures among orthopedic physicians. Furthermore, many studies focus on one or a small number of measures, and compliance rates are often not reported, especially in regard to overall compliance, i.e., being compliant with multiple measures [14]. This is unfortunate given evidence from abdominal surgery that larger bundles of preventive measures are most effective in terms of SSI reduction [20]. Thus, it remains both important and challenging to estimate overall compliance and its determinants, resulting in a disadvantageous research gap because these issues are crucial to better understand how to promote complex compliance bundles.

Against this background, this study will report data on psychosocial correlates of self-reported compliance with 26 SSI preventive measures among orthopedic physicians participating in the pilot survey of the WACH-trial (“Wundinfektionen und Antibiotikaverbrauch in der Chirurgie” [Wound Infections and Antibiotics Consumption in Surgery] [21]). Since reporting compliance with multiple measures already represents a time-consuming task for study participants, it was necessary to use a parsimonious behavioral theory. Thus, the COM-B (Capability, Opportunity, Motivation and Behavior) model [22] was selected, which—while being designed to integrate numerous theoretical constructs [23]—conceives behavior to be determined by three basic components: capability, opportunity, and motivation. Additional file 1: Table S1 provides their definitions, and expands on specific examples regarding SSI-preventive measures. In sum, this study aims at an initial assessment of orthopedic physicians’ SSI-preventive self-reported compliance with multiple measures and its associations with COM-B-delineated psychosocial determinants.

## Methods

### Design, setting, and study participants

A cross-sectional questionnaire survey was conducted as a pilot for the WACH-trial (German Clinical Trials Register-ID: DRKS00015502) [21] from January 28th to March 1st 2019, in a tertiary care university orthopedic clinic in Hannover, Germany. The clinic is both affiliated to Hannover Medical School and the non-profit hospital group DIAKOVERE Ltd., and has five elective operating theaters and five general wards. The survey was approved by Hannover Medical School’s ethics committee (No. 8219_BO_K_2018), its employees’ council, its data protection office, and the employee representation at DIAKOVERE Annastift as the orthopedic clinic’s operator. All orthopedic surgeons (n = 50) and anesthesiologists (n = 21) were invited to participate in the survey. The questionnaires were distributed primarily by the secretaries, and after self-administration were returned via sealed collection boxes. To stimulate a high survey response, incentives (10 × 2 one-day wellness vouchers for a local spa club) were raffled among all participants.

### Measures

The questionnaire included items to assess the respondents’ knowledge of existing clinic specific standards regarding SSI preventive measures, their estimation of their compliance with measures, compliance determinants (COM-B), interventions in the clinic to promote compliance (as perceived by respondents), and professional and sociodemographic characteristics. In the following, items used in this study will be presented.

#### Self-reported SSI preventive compliance

A total of 26 SSI preventive measures (see Table 1) were selected based on the most recent SSI prevention recommendation by the German Commission on Hospital Hygiene and Infection Protection at the Robert Koch-Institute (KRINKO) and the respective guideline by the German Association of the Scientific Medical Societies (AWMF) [24, 25]. For each preventive measure, the survey participants were asked to indicate the number of instances in which they, to their own assessment, executed each measure compliantly (as a percentage of those instances where the measure is recommended). If participants considered specific measures not to fall within their area of responsibility, the answer category “not applicable” was offered. An index to determine overall compliance for each participant was algorithmized as follows: All measure-specific self-reported compliance rates a given participant had indicated were summed up and divided by the number of measures he or she had indicated responsibility for. To adjust analyses of this “overall compliance”-index for the quantity of measures a respondent reported responsibility for, a count variable “responsibility” was created based on the number of such measures he or she indicated.

**Table 1:** Self-reported SSI preventive compliance rates, in descending order by compliance

Preventive measure	Number (%) of respondents with self-reported responsibility for the measure	Mean compliance rate	SD
Wearing surgical cap	49 (94.2%)	100%	0.1
Use of double gloving	35 (67.3%)	100%	0.0
Preparing of sterile instruments within the operating theatre	19 (36.5%)	99.5%	2.3
Sterile handing over of instruments in the operating theatre	33 (63.5%)	98.9%	2.7
Wearing surgical mask	49 (94.2%)	98.3%	7.7
Covering prepared sterile instruments within the operating theatre	17 (32.7%)	97.1%	6.6
Using remnant antiseptic	38 (73.1%)	96.9%	6.4
Hygienic hand disinfection after exposure to potentially infectious material	52 (100%)	96.5%	6.6
Preparing of sterile instruments outside the operating theatre	27 (51.9%)	96.3%	8.3
Perioperative antibiotic prophylaxis	39 (75.0%)	95.9%	10.0
Surgical hand disinfection—technique	45 (86.5%)	95.0%	12.5
Examination of the indication of existing surgical drains	34 (65.4%)	93.4%	14.9
Use of iodine-impregnated incision drape	29 (55.8%)	93.1%	15.1
Surgical hand disinfection—exposure time	44 (84.6%)	93.0%	13.7
Perioperative temperature measurement	22 (42.3%)	91.6%	17.8
Hygienic hand disinfection before aseptic procedures	52 (100%)	91.3%	13.9
Hygienic hand disinfection after touching a patient	52 (100%)	87.4%	14.6
Aseptic dressing change	40 (76.9%)	86.1%	24.1
Perioperative blood glucose control	23 (44.2%)	83.5%	27.4
Perioperative pre-warming	22 (42.3%)	81.8%	27.2
Septic dressing change	35 (67.3%)	81.5%	29.3
Covering prepared sterile instruments outside the operating theatre	16 (30.8%)	80.6%	35.8
Hair removal—clipping	15 (28.8%)	79.3%	26.3
Hygienic hand disinfection before touching a patient	52 (100%)	78.7%	21.5
Hygienic hand disinfection after touching patients surroundings	52 (100%)	72.0%	24.0
Removing white coat before touching a patient^a^	43 (82.7%)	53.8%	34.5
Mean overall compliance rate (index)	52	88.9%	7.9

SD, standard deviation

^a^This refers to situations outside the operating theatre, where physicians in this clinic wear white trousers and white short-sleeved shirts plus a white long-sleeved coat (all provided for by the clinic), the latter of which is recommended to be removed before touching a patient (especially before activities such as aseptic dressing changes and redon drains, in which cases one is supposed to change to a single use protective coat)

#### Psychosocial determinants of SSI preventive compliance

For item development, previous COM-B-publications were screened [18, 19, 26–30]. Eventually, items for every COM-B subcategory were included in the questionnaire: physical capability (2 items), psychological capability (4 items), physical opportunity (3 items), social opportunity (3 items), reflective motivation (5 items), and automatic motivation (1 item; see Additional file 2: Table S2 and Additional file 3: Table S3). In all items, the expression “these measures” referred to those SSI preventive measures that respondents self-reported to be responsible for. Seven-point Likert scales were used (1 “does not apply at all”—7 “does apply completely”). To determine the empirical structure underlying these items, one explorative principal-components factor analysis using oblique rotation with Kaiser Normalization was conducted for motivation and capability items, which assess personal attributes, and one analysis was conducted for the opportunity items, which assess environmental facilitators and barriers. As Table S2 shows for the former item set, three factors, which each explained at least 10% of the total variance, emerged and were termed capabilities, motivation, and planning (the item “I regularly make sure that I implemented these measures correctly” was omitted since it did not load above 0.5 on any one of the three factors). As Table S3 shows for the opportunity items, only one factor emerged, on which all six items loaded higher than 0.5. Cronbach’s alphas for all four resulting scales exceeded 0.8 (for details, see Tables S2 and S3).

#### Sociodemographic characteristics and professional groups

Respondents were asked to indicate their sex, age (for data protection reasons in classified format: < 18, 18–30, 31–40, 41–50, 51–60, > 60 years), and their profession in the clinical context (specialist for orthopedics and trauma surgery, specialist for anesthesiology, further training assistant for orthopedics and trauma surgery, and further training assistant for anesthesiology; corresponding categories for nurses were included as well, but as the survey response rate among nurses was 17.3%, only the results for physicians are reported here). These professional categories were summarized into orthopedic surgeons and anesthesiologists.

### Statistical analysis

In addition to descriptive and bivariate correlational analyses, a backward linear regression analysis was conducted to scrutinize the specific relationships between self-reported overall compliance and the hypothesized determinants, i.e., motivation, capabilities, opportunities, and planning. In this analysis, type of physician and the number of preventive measures that respondents self-reported to be responsible for (variable termed “responsibility”) were adjusted for. To visualize significant associations with COM-B factors, the eventually identified determinants were trichotomized into low, medium, and high scores, and estimated means of the overall compliance index were plotted with error bars (standard errors). Analyses were conducted using IBM^©^ SPSS^©^ Statistics (version 26).

## Results

### Sample description

Fifty-two physicians took part in the pilot survey, corresponding to a response rate of 73.2%. Of these, 32.7% were women, and 73.1% were orthopedic surgeons. Regarding age, 23.1% were 18–30 years old, 30.8% were 31–40 years old, 23.1% were 41–50 years old, 19.2% were 51–60 years old, and 3.8% were older than 60 years.

### Univariate distributions

In Tables S2 and S3, the mean values for the COM-B-delineated items and scales are shown. The highest mean pertained to the factor “motivation”; in particular, the sense of obligation to permanently implement the measures correctly is very prevalent with an average of 6.5. Additionally, the conviction that the correct application will contribute to the prevention of SSI and the goal to always implement those measures correctly are prominent with an average value of 6.3. In addition, the items defining the capability factor were relatively highly rated, with mean values ranging from 6.1 to 5.5. The items related to the opportunity factor, which among other things included the perception of the available technical and spatial equipment and the sufficiency of recognition received for implementing SSI preventive measures, were rated lower than the motivation and capability items, with mean values ranging from 5.5 to 4.1. While still lying above the scale’s midpoint, the planning factor was associated with the lowest ratings. Planning how to implement the SSI preventive measures most effectively (action planning) and planning how to deal with barriers (coping planning) received mean ratings of 4.5 and 4.0, respectively, while the scale mean was 4.3.

As Table 1 shows, the mean self-reported overall compliance rate, i.e., averaged across all 26 preventive measures, was 88.9%. Regarding individual measures, for four measures (hair removal/clipping, hygienic hand disinfection before touching a patient, hygienic hand disinfection after touching patient’s surroundings, and removing white coat before touching a patient), a mean self-reported compliance rate less than 80% was reported, while rates of over 90% were obtained for 16 measures. While hygienic hand disinfection was the only behavioral domain that all respondents reported as their own responsibility (with compliance rates across indications ranging from 96.5% to 72%), all other measures were seen as part of one’s own tasks by varying rates of physicians. These ranged from 94.2% for wearing surgical masks and hoods to covering prepared sterile instruments within and outside the operating theatre and hair removal by clipping, which were reported as their tasks by less than a third of respondents.

### Correlation analysis

Bi-variately, self-reported overall compliance was positively associated with all COM-B factors, and positively but only marginally significantly associated with type of physician (reflecting rates of 90% for surgeons and 85.9% for anesthesiologists). As Table 2 further shows, while the association with motivation was modest (r = 0.27), higher coefficients were obtained for planning (r = 0.38), opportunities (r = 0.42), and capabilities (r = 0.51). Other correlations included positive but only partly significant coefficients of the responsibility-index with the COM-B-factors and invariably significant associations within the latter, ranging from r = 0.28 for motivation/opportunities to r = 0.59 for planning/opportunities.

**Table 2:** Bivariate correlations of type of physician, self-reported overall compliance rate, responsibility, motivation, capabilities, opportunities and planning

		Overall Compliance	Responsibility	Motivation	Capabilities	Opportunities	Planning
Type of Physician (1 = s.**, 0 = a.***)	rpb*	0.23	0.18	− 0.24	0.21	0.09	-0.14
	p	= 0.097	= 0.209	= 0.776	= 0.141	= 0.540	= 0.333
	N	52	52	52	51	51	50
Overall Compliance	r****		0.12	0.27	0.51	0.42	0.38
	p		= 0.411	= 0.051	< 0.001	= 0.002	= 0.007
	N		52	52	51	51	50
Responsibility	r			0.24	0.31	0.22	0.37
	p			= 0.086	= 0.025	= 0.127	= 0.009
	N			52	51	51	50
Motivation	r				0.40	0.28	0.32
	p				= 0.004	= 0.044	= 0.024
	N				51	51	50
Capabilities	r					0.57	0.43
	p					< 0.001	= 0.002
	N					50	50
Opportunities	r						0.59
	p						< 0.001
	N						49

*rpb = point biserial correlation coefficient; **s. = surgeon; ***a. = anesthesiologist; ****r = Pearson correlation coefficient

### Regression analysis

**Fig. 1:**
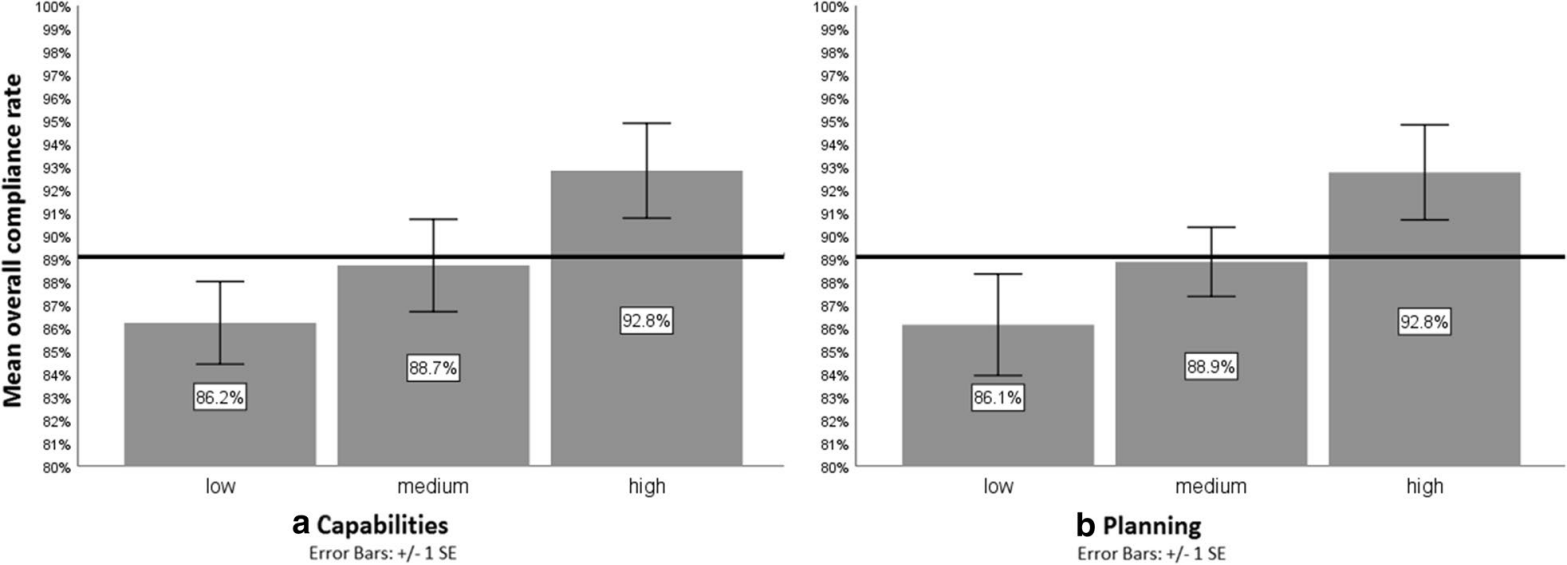
Mean self-reported SSI-preventive overall compliance rates (in %) by **a** “capabilities” and **b** “planning” (both as trichotomized scales) (black line represents the grand mean of overall compliance)

Table 3 shows the results of the backward linear regression modeling for the overall compliance index. In the first model, i.e., mutually adjusting for all regressors, of the COM-B factors capabilities and planning showed a specific effect on compliance, respectively. After omitting predictors in the subsequent models (probability to remove: 0.09), both capabilities and planning retained their effect. As Fig. 1 shows, the mean overall compliance rate in subgroups defined by these two determinants was significantly higher than the grand mean only given high levels of capabilities and planning, respectively.

**Table 3:** Results of backward linear regression model with self-reported overall compliance rate as regressand, and type of physician (surgeon or anesthesiologist), responsibility, motivation, capabilities, opportunities and planning as regressors

		Model 1	Model 2	Model 3	Model 4
(Constant)	t	7.71	9.90	9.99	9.68
	p	< 0.001	< 0.001	< 0.001	< 0.001
Surgeon (reference category: anesthesiologist)	β	0.30	0.29	0.28	0.22
	t	2.23	2.22	2.21	1.75
	p	= 0.031	= 0.032	= 0.032	= 0.086
Responsibility	β	− 0.24	− 0.24	− 0.23	
	t	− 1.76	− 1.74	− 1.71	
	p	= 0.086	= 0.090	= 0.095	
Motivation	β	0.05			
	t	0.37			
	p	= 0.711			
Capabilities	β	0.39	0.41	0.38	0.35
	t	2.45	2.68	2.80	2.54
	p	= 0.019	= 0.010	= 0.008	= 0.015
Opportunities	β	− 0.06	− 0.07		
	t	− 0.38	− 0.39		
	p	= 0.707	= 0.698		
Planning	β	0.38	0.38	0.35	0.29
	t	2.27	2.37	2.54	2.11
	p	= 0.028	= 0.023	= 0.015	= 0.041

β = standardized regression coefficient, t = t statistic (unstandardized regression coefficient divided by standard error)

## Discussion

Results can be summarized as follows. First, based on 18 items to assess COM-B-determinants of SSI preventive compliance of orthopedic physicians, a specific factor for their action and coping planning of preventive measures emerged. In addition, while among the COM-B factors, motivation was rated highest, followed by capabilities, opportunities, and planning, self-reported compliance rates ranged from complete implementation (wearing surgical cap and using double gloving) to just above 50% (removing white coat before touching a patient). Furthermore, in bivariate analysis, overall compliance correlated highest with capabilities, followed by opportunities, planning, and motivation. Finally, in multiple regression analysis, specific associations with compliance were found for capabilities and planning.

Before further discussion, the strengths and limitations of the present study must be considered. First, our response rate (73%) exceeds that of other surveys of physicians working in German hospitals, e.g., in the study most comparable to the present field time (first quarter of 2019), the response rate was 54% among thoracic surgeons [31]. At the same time, it does match our own experience with surveying physicians at Hannover Medical School (71%) [32, 33]. Second, the assessment of virtually all recommended SSI preventive measures with simultaneous operationalization of psychosocial determinants based on one behavioral theory is, to our knowledge, unprecedented in orthopedic surgery.

Turning to the study’s limitations, first it is cross-sectional by design, and thus reverse causation cannot be ruled out. For instance, it is possible that a person who perceives his or her compliance to be high will, in hindsight, rate his or her capabilities and planning efforts to be high as well. Nevertheless, reporting associations between self-reported SSI preventive compliance and psychosocial factors suggested by state-of-the-art behavioral theory in an orthopedic physician survey with an above-average response rate was considered to be warranted.

Second, the study was confined to self-reported compliance, with relatively high compliance levels found. In part, this may be due to the clinic being confined to elective surgery, and the intensive infection prevention and control cooperation with Hannover Medical School’s hygiene and hospital epidemiology unit. Furthermore, self-reported behavior does not necessarily correlate with and correspond to observed behavior, which has been linked to overconfidence in recent studies on hand hygiene [34–36]. However, on the one hand, data on this behavioral domain from tertiary care hospitals in Germany did find significant positive correlations between self-reported and observed compliance (r = 0.55) [37], which may be due to improved realistic confidence in hospitals with an extensive history of infection prevention interventions. On the other hand, if “… people believe that their hand hygiene is much better than it is, they are likely to be oblivious to current campaigns that aim to increase hand hygiene behavior by changing attitude” [34, p. 421]. Thus, behavior change techniques such as “incompatible beliefs” (i.e., drawing attention to discrepancies [38]) could use subjective compliance estimates as a reference of comparison when providing feedback on behavior.

Third, items for compliance determinants were phrased to relate to all SSI preventive measures that respondents felt responsible for, precluding insights for differences in determinants across measures. This was accepted to ensure a feasible survey instrument, since assessment of every determinant item for each individual measure would have implied 486 items (vs. 44 items in the instrument as deployed).

Finally, with N = 52, the sample, being from a pilot survey, was small. This also prevented analyses stratified for type of physician, i.e. surgeons vs. anesthesiologists, and the tendency for surgeons reported higher compliance should be treated with caution. However, considering the response rate, the survey in our view does provide a valid grasp of compliance determinants with regard to SSI preventive measures in the specific clinic in which it was conducted.

Keeping these limitations in mind, the results can be rationalized as follows. First, items for which respondents rated their confidence in their capability to implement SSI preventive measures, and thus would originally pertain to motivation [39], emerged as aspects of capabilities in the current data. In this regard, it might be instructive to note that self-efficacy, i.e., one’s confidence in one’s own capability to perform a given behavior, has been differentiated into motivational self-efficacy, which refers to goal-setting, i.e., choosing behaviors, and volitional self-efficacy, which refers to the pursuit of goals, i.e., implementing behavior [40]. Since the present two confidence items referred to this latter step, in which behavioral skills are more important than goal contemplation, their loadings on the capabilities-factor may indicate that not all types of self-efficacy are necessarily motivational.

Second, the finding that motivation was significantly associated with compliance bi-variately only, i.e. not when adjusting for other COM-B factors, can be explained by capabilities and planning mediating this association. In contrast, further research is needed in regard to opportunities, i.e. an in-depth analysis of the interplay of psychological and environmental factors (e.g. in terms of effect modifications) was beyond the scope of the present pilot study.

Finally, the associations found between physicians’ assessments of their SSI-preventive capabilities and planning with their self-reported compliance provide (albeit, given the limitations of this pilot study, unequivocally tentative) hints regarding specific approaches in promoting SSI preventive compliance in orthopedic surgery. While implementation interventions such as educational training sessions, which integrate skills and capabilities, are quite common in SSI prevention (besides “pure” education) [14, 41, 42], components to promote planning skills are used less so far (consistent with the lowest rating for “planning” among all COM-B-factors in the present sample). In particular, planning skills may contribute to overcoming the so-called intention-behavior gap, i.e., situations in which healthcare workers intend to enact a certain behavior, but eventually do not [43, 44]. However, as interventions to encourage action and coping planning in earlier studies were successful in regard to nurses only [18, 45, 46], interventions for physicians have yet to be developed.

## Conclusion

In sum, this study provides quite preliminary but yet theoretically meaningful and potentially instructive insight into the psychology of SSI prevention as perceived by orthopedic surgeons. In the scrutinized clinic, this professional group rated their SSI preventive compliance as high and reported to be motivated and capable in this regard, but seemed to have potential for developing relevant planning skills. High capabilities and planning scores were associated with higher self-reported compliance. Due to the small sample size and the specificities of the pilot survey setting, further research is needed to test whether the results are generalizable to other hospitals. As of March 2021, data of the multicenter, parallel-group, cluster-randomized controlled WACH-trial are being analyzed, and evidence generated on effects of interventions addressing these compliance determinants via hospital infection prevention and control teams [21].

## Supplementary Information

**Additional file 1: Table S1.** Overview of COM-B factors.

**Additional file 2: Table S2.** Items targeting the capability and the motivation components of the COM-B model.

**Additional file 3: Table S3.** Items targeting the opportunity component of the COM-B model.

## Data Availability

The datasets used and/or analyzed during the current study are available from the corresponding author on reasonable request.

## Abbreviations

AWMF: Arbeitsgemeinschaft der Wissenschaftlichen Medizinischen Fachgesellschaften e.V. [German Association of the Scientific Medical Societies]
COM-B: Capability, Opportunity, Motivation and Behavior
DRKS: Deutsches Register Klinischer Studien [German Clinical Trials Register]
KRINKO: Kommission für Krankenhaushygiene und Infektionsprävention [German Commission on Hospital Hygiene and Infection Protection at the Robert Koch Institute]
SSIs: Surgical site infections
WACH: Wundinfektionen und Antibiotikaverbrauch in der Chirurgie [Wound Infections and Antibiotics Consumption in Surgery] = trial acronym
