# Supplementary material for: Determinants of orthopedic physicians’ self-reported compliance with surgical site infection prevention: results of the WACH-trial’s pilot survey on COM-B factors in a German university hospital
Source: Antimicrob Resist Infect Control. 2021 Apr 7;10:67. doi: 10.1186/s13756-021-00932-9 (PMC8025554; doi:10.1186/s13756-021-00932-9)
Supplement: Supplementary file 1 — Additional file 1: Table S1. Overview of COM-B factors. [file 13756_2021_932_MOESM1_ESM.docx]

**Table S1:** Overview of COM-B factors

| **COM-B factor** | **Definition*** | **Generic examples* (specific examples, i.e. for compliance with  SSI-preventive measures)** |
| --- | --- | --- |
| Capability | Physical and psychological capacity to engage in and execute a given behavior or activity. | Knowledge, skills (know how to correctly wear a surgical mask; be able to implement surgical hand disinfection according to guidelines) |
| Motivation | Reflective (e.g. cognitive) and automatic (e.g. habitual) processes that energize and direct a given behavior or activity. | Analytical decision-making and goal setting, emotional responses, habitual processes (setting the goal to correctly disinfecting one’s hands before every aseptic procedure; being socially acknowledged for compliant aseptic dressing change) |
| Opportunity | Factors that lie outside of the individual and make a given behavior or activity possible, or prompt it. | Facilitators, barriers (support by leaders and superiors; lack of hand sanitizer dispensers) |

*following ^22^
