# Supplementary material for: Determinants of orthopedic physicians’ self-reported compliance with surgical site infection prevention: results of the WACH-trial’s pilot survey on COM-B factors in a German university hospital
Source: Antimicrob Resist Infect Control. 2021 Apr 7;10:67. doi: 10.1186/s13756-021-00932-9 (PMC8025554; doi:10.1186/s13756-021-00932-9)
Supplement: Supplementary file 2 — Additional file 2: Table S2. Items targeting the capability and the motivation components of the COM-B model. [file 13756_2021_932_MOESM2_ESM.docx]

**Table S2:** Items targeting the capability and the motivation components of the COM-B model: (a) results of the factor analysis (pattern matrix, oblique rotation^#^), (b) means and standard deviations (SD) of items and scales*

|  | **(a)** | | | **(b)** | | |
| --- | --- | --- | --- | --- | --- | --- |
| **Items** | **Capability**  (Eigenvalue**: 4.9; variance explained: 44.8%) | **Motivation** (Eigenvalue: 1.9; variance explained: 17.7%) | **Planning** (Eigenvalue: 1.3; variance explained: 11.8%) | **N** | **Mean***** | **SD** |
| I feel confident to be capable of implementing these measures in practice correctly.) | .96 |  |  | 52 | 5.9 | 0.98 |
| I have been sufficiently trained to implement these measures in accordance with guidelines. | .85 |  |  | 52 | 5.5 | 1.34 |
| I know exactly how to implement these measures correctly. | .84 |  |  | 51 | 5.6 | 1.25 |
| I feel confident to be implementing these measures correctly again after committing an error. | .77 |  |  | 52 | 6.1 | 0.89 |
| The effective implementation of these measures is routine for me. | .62 |  |  | 52 | 5.6 | 1.19 |
| I regularly make sure that I implemented these measures correctly.^+^ |  |  |  | 52 | 5.7 | 1.18 |
| **Scale “Capabilities”** (Cronbach’s alpha: 0.89) |  |  |  | 51 | 5.8 | 0.95 |
| As an employee, I feel obliged to implement these measures correctly at all times. |  | .88 |  | 52 | 6.5 | 0.87 |
| If I implement these measures correctly, I will contribute to the prevention of surgical site infections. |  | .83 |  | 52 | 6.3 | 1.08 |
| If I implement these measures correctly, I will be a role model for my colleagues. |  | .76 |  | 52 | 5.9 | 1.44 |
| I have set myself the goal of always implementing these measures correctly. |  | .75 |  | 52 | 6.3 | 0.96 |
| **Scale “Motivation”** (Cronbach’s alpha: 0.83) |  |  |  | 52 | 6.3 | 0.90 |
| I have recently planned how to implement these measures most effectively. |  |  | .95 | 51 | 4.5 | 1.60 |
| I have recently planned how to deal with barriers and events, which make it difficult for me to implement these measures correctly. |  |  | .85 | 51 | 4.0 | 1.68 |
| **Scale “Planning”** (Cronbach’s alpha: 0.82) |  |  |  | 50 | 4.3 | 1.52 |

Notes: ^#^ For bivariate correlations of resulting scale with other components, see Table 2; *Factor loadings <.50 omitted; **Factors with an Eigenvalue ≥1 explain more variance than a single observed variable; ***Mean value on the Likert scale (1 “does not apply at all” - 7 “does completely apply”)
