# Supplementary material for: Determinants of orthopedic physicians’ self-reported compliance with surgical site infection prevention: results of the WACH-trial’s pilot survey on COM-B factors in a German university hospital
Source: Antimicrob Resist Infect Control. 2021 Apr 7;10:67. doi: 10.1186/s13756-021-00932-9 (PMC8025554; doi:10.1186/s13756-021-00932-9)
Supplement: Supplementary file 3 — Additional file 3: Table S3. Items targeting the opportunity component of the COM-B model. [file 13756_2021_932_MOESM3_ESM.docx]

**Table S3:** Items targeting the opportunity component of the COM-B model: (a) results of the factor analysis (component matrix^§,#^), (b) means and standard deviations (SD) of items and scale*

|  | **(a)** | **(b)** | | | |
| --- | --- | --- | --- | --- | --- |
| **Items** | **Opportunity**  (Eigenvalue**: 3.8;  variance explained: 62.5%) | **N** | **Mean***** | **SD** |  |
| I have sufficient time to implement these measures effectively. | .89 | 52 | 4.5 | 1.71 |  |
| The personnel resources on “my” ward and in the operating theatre are appropriate to implement these measures. | .88 | 51 | 4.6 | 1.79 |  |
| The technical and spatial equipment on “my” ward and in the operating theatre is appropriate to implement these measures. | .82 | 52 | 5.5 | 1.57 |  |
| Cooperation with my colleagues on these measures is working well. | .79 | 52 | 5.4 | 1.29 |  |
| I get enough opportunities to inform myself about the correct implementation of these measures (in-house standards). | .77 | 52 | 5.0 | 1.53 |  |
| I receive sufficient recognition for the implementation of these measures, e.g. from my superiors. | .55 | 52 | 4.1 | 1.79 |  |
| **Scale “Opportunities”** (Cronbach’s alpha: 0.87) |  | 51 | 4.9 | 1.27 |  |

Notes: ^§^Only one factor explained at least 10% of the total variance; ^#^ For bivariate correlations of resulting scale with other components, see Table 2; *Factor loadings <.50 omitted; **Factors with an Eigenvalue ≥1 explain more variance than a single observed variable; ***Mean value on the Likert scale (1 “does not apply at all” - 7 “does completely apply”)
